# Association between triglyceride-glucose index and its obesity indicators with hypertension in postmenopausal women: a cross-sectional study

**DOI:** 10.3389/fnut.2025.1623697

**Published:** 2025-07-17

**Authors:** Bo Zhang, Daoli Jiang, He Ma, Huanxian Liu

**Affiliations:** ^1^Department of Pharmacy, The Affiliated Hospital of Xuzhou Medical University, Xuzhou, Jiangsu, China; ^2^Department of Medical Record and Statistics, The Affiliated Hospital of Xuzhou Medical University, Xuzhou, Jiangsu, China; ^3^Department of Neurology, Chinese PLA General Hospital, Beijing, China

**Keywords:** metabolic biomarkers, obesity, hypertension, postmenopausal health, nutrition

## Abstract

**Background:**

This study explored the association between the triglyceride-glucose (TyG) index, combined with adiposity metrics, and hypertension prevalence in postmenopausal women.

**Methods:**

A cross-sectional study was conducted using data from 4,302 postmenopausal women in the National Health and Nutrition Examination Survey (NHANES) from 1999 to 2018. Multivariable adjusted logistic regression models and restricted cubic splines (RCS) were implemented to assess the dose-response relationship. Receiver operating characteristic (ROC) curves were employed to compare the diagnostic performance of the TyG index, TyG-body mass (TyG-BMI), TyG-waist circumference (TyG-WC), and TyG-waist-to-height ratio (TyG-WHtR).

**Results:**

Multivariable-adjusted analyses demonstrated that the TyG index and its obesity indicators are significantly associated with hypertension risk. The RCS curve exhibited a non-linear relationship between TyG-WHtR and hypertension (P for non-linearity = 0.026), whereas other indices showed linear associations. ROC analysis confirmed the superior discriminative ability of TyG-WHtR for hypertension (AUC = 0.643, 95% CI 0.625–0.660).

**Conclusion:**

The TyG index and its combined obesity indicators, particularly TyG-WHtR, are strongly associated with hypertension risk in postmenopausal women. TyG-WHtR may serve as a valuable biomarker for targeted screening in this population.

## Introduction

As a prevalent and clinically significant cardiovascular disorder, hypertension is a global health challenge that causes significant morbidity in all populations (1). Postmenopausal women are particularly vulnerable to hypertension due to decreased endogenous estrogen protection and age-related homeostatic changes resulting from the cessation of ovarian function. These changes include a reduction in estradiol and an increase in follicle-stimulating hormone (FSH) (2). Moreover, the onset of menopause is associated with increased oxidative stress, which can impair vascular function and promote inflammation, thereby further elevating the risk of hypertension development (3). Consistent with these findings, epidemiological studies have shown that postmenopausal women have a higher prevalence of hypertension compared to premenopausal women and age-matched men (4–6).

The pathophysiological nexus between insulin resistance (IR) and hypertension has been comprehensively characterized, with IR-driven endothelial dysfunction and sympathetic activation serving as pivotal mediators (7). The hyperinsulinemic-euglycemic clamp (HEC) persists as the criterion-reference methodology for precise quantification of insulin resistance (IR) in clinical metabolic research; however, this technique is considered to be complicated in application and intrusive (8). The homeostasis model assessment of insulin resistance (HOMA-IR) provides a simplified alternative, yet its clinical application is also limited by the requirement of fasting insulin level measurement (9). The triglyceride-glucose (TyG) index, derived from the fasting triglyceride and fasting plasma glucose levels, has emerged as a practical surrogate with validated diagnostic accuracy (10). Recent studies have demonstrated the TyG index is associated with a number of cardiometabolic outcomes, including hypertension (11–13), yet its prognostic utility specifically in postmenopausal women remains limited.

Obesity has been demonstrated to be closely linked to the development of insulin resistance and hypertension (14). Postmenopausal women have been shown to be particularly susceptible to weight gain, with a prevalence of obesity reaching as high as 40%. This demographic also exhibits a distinct pattern of adiposity distribution, characterized by a greater amount and more concentrated accumulation of visceral fat compared to premenopausal women (15). Recent studies have indicated that the combination of the TyG index with obesity indicators, particularly body mass index (BMI), waist circumference (WC), and waist-to-height ratio (WHtR) is more effective for predicting hypertension (16, 17).

Therefore, the present study leverages nationally representative National Health and Nutrition Examination Survey (NHANES) data to explore the potential association between variations in the TyG index, particularly when integrated with adiposity metrics, and the prevalence of hypertension in postmenopausal cohorts, with the aim of providing key evidence for a targeted screening tool for hypertension in this population to promote early intervention of the disease and improve clinical outcomes.

## Materials and methods

### Data source

National Health and Nutrition Examination Survey, coordinated through the National Center for Health Statistics (NCHS) (18), aims to evaluate the health and nutritional status of the United States population. Its stratified multistage probability sampling methodology ensures that each survey cycle produces a sample representative of the whole country (19). The survey is divided into two parts: a household interview and a medical examination at a Mobile Examination Center (MEC). Ethical oversight for NHANES was administered by the NCHS and all individuals providing informed consent.

**FIGURE 1:**
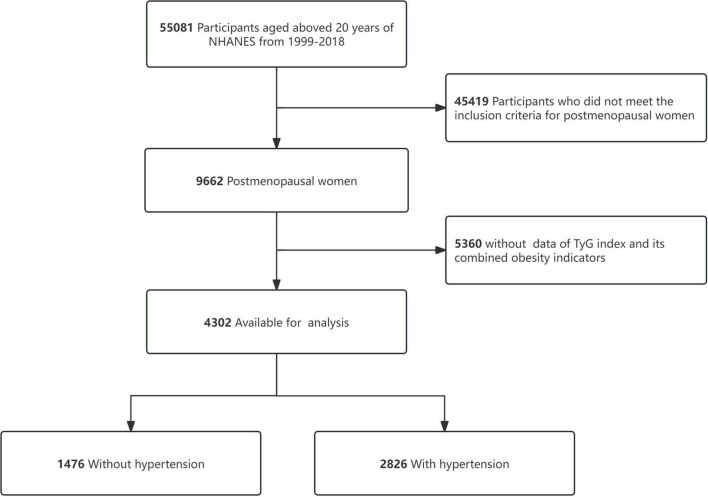
Study flow chart.

This investigation utilized data spanning ten NHANES survey cycles (1999–2018), with rigorous exclusion protocols applied to ensure an accurate sample size. Exclusion criteria for the analysis included participants who did not meet the inclusion criteria for postmenopausal women (*n* = 45,419) and missing data for the TyG index and its related adiposity metrics (*n* = 5,360). The analysis framework included 4,302 eligible participants, as depicted in Figure 1.

### Assessment of TyG index and TyG-related indices

The TyG index and its associated indices in the study were defined and calculated as follows: BMI was determined by weight (kg)/height squared (m^2^), while WC was the waist circumference (cm), and WHtR was determined by WC (cm)/height (cm). TyG index = ln [fasting triglyceride (mg/dL) × fasting glucose (mg/dL)/2]; TyG-WC = TyG × WC; TyG-WHtR = TyG × WHtR; TyG-BMI = TyG × BMI.

### Assessment of menopausal status

To determine menopausal status in this study, we used a two-step questionnaire from the NHANES Reproductive Health Questionnaire. First, participants were asked whether they had experienced at least one menstrual period in the past 12 months. They were subsequently inquired about the reason for not having menstruated in the previous 12 months. Women were classified as postmenopausal if they responded “no” to the first question and indicated either “menopause” or “hysterectomy” in response to the second question.

### Diagnosis of hypertension

Hypertension was defined as meeting any of the subsequent conditions: (1) an average systolic blood pressure of ≥ 140 mmHg and/or an average diastolic blood pressure of ≥ 90 mmHg according to the guidelines established by the International Society of Hypertension; (2) self-reported physician-diagnosed hypertension; or (3) current use of prescription antihypertensive medications.

### Covariate data collection

In our analysis, we controlled for several potential confounders, guided by existing literature and clinical expertise, including sociodemographic factors such as age, racial/ethnic categorization, marital status, educational attainment, and family income; behavioral modifiers including smoking, drinking habits, and physical activity; Assayed variables included high-density lipoprotein cholesterol (HDL-C), low-density lipoprotein cholesterol (LDL-C), total cholesterol (TC), glycosylated hemoglobin (HbA1c), and uric acid; and finally, characteristics of menopausal metabolic consisted of menopausal age and hormone replacement therapy. Marital status was divided into two groups: married or living with a partner and living alone. Race categories included Mexican American, Non-Hispanic Black, Non-Hispanic White, other Hispanic, and other races (20). Educational attainment was categorized into three levels: less than high school, high school or equivalent, and more than high school (21). Family income was stratified into three groups by the poverty income ratio (PIR): below 1.3, between 1.3 and 3.5, and above 3.5. Physical activity was determined by the amount of time (in minutes) spent in different activities per week. Smoking status was categorized as never smoker (smoked < 100 cigarettes), current smoker, and former smoker (quit smoking after smoking ≥ 100 cigarettes). Drinking status was divided into never drinker (lifetime alcohol consumption < 12 drinks), former drinker (≥ 12 drinks in a year but abstained in the past year, or no drinks last year but ≥ 12 lifetime drinks), and current drinker. Menopausal age was considered as a continuous variable in the Cox regression analysis. The determination of hormone replacement therapy (HRT) was derived from participants’ questionnaire responses indicating past use of female hormones, including estrogen and progesterone.

### Statistical analysis

This study was a secondary analysis of publicly available datasets. Baseline characteristics of the study participants were stratified by hypertension status and compared using appropriate statistical tests. Due to the percentages of missing values being found to be less than 20%, multiple imputations were used to impute missing data for the covariates, resulting in the generation of five independent datasets that were subsequently evaluated collectively; detailed information regarding multiple imputation is provided in the supplementary methods. Descriptive statistics were used to describe the baseline characteristics; categorical data were expressed as frequencies and percentages, while continuous data were expressed as means ± SD and medians (interquartile range), taking into account any skewed data distribution. Normality was assessed using the Shapiro-Wilk test to validate the selected statistical methods. Chi-squared analyses were used for categorical variables, and analysis of variance or Mann-Whitney U tests were used for continuous variables.

To investigate the independent and combined effects of the TyG index and its integration with adiposity metrics (TyG-BMI, TyG-WC, and TyG-WHtR) on the prevalence of hypertension among postmenopausal women, we implemented multivariable logistic regression modeling. We standardized (Z-score) the TyG index and its obesity indicators, then included them in the multivariable logistic analyses. Additionally, we categorized the continuous variables into tertiles, with the first tertile of the TyG index and its obesity indicators serving as the reference. Odds ratios (ORs) with 95% confidence intervals (CIs) were computed per standard deviation (SD) increment and across tertiles (T1–T3) of the TyG index and its associated indices. Four hierarchical models were constructed to adjust for confounders: Model 1 was the crude model, not accounting for any covariates. Model 2 was adjusted for age, race/ethnicity, marital status, education level, PIR, and NHANES cycles. Model 3 was additionally adjusted for smoking, drinking status, and physical activity. Model 4 included all preceding variables augmented along with HDL-C, LDL-C, HbA1c, uric acid, menopausal age, and hormone replacement therapy. When analyzing dose-response gradients across tertile divisions, median values within each stratified subgroup were operationalized as continuous parameters.

To investigate the potential curvilinear associations, restricted cubic spline (RCS) modeling with three knots was implemented, complemented by likelihood ratio tests examining the goodness-of-fit.

The classification accuracy of TyG-related indices was assessed through the implementation of a receiver operating characteristic (ROC) framework. The discriminative ability of these indices was quantified by calculating the area under the curve (AUC) metrics, with 1,000 bootstrap-resampled iterations employed to enhance the robustness of the estimates. Intergroup comparisons were conducted via two-sample independent *t*-test procedures.

To assess the validity of TyG-related indices, we conducted sensitivity analyses using multivariate regression and ROC curves to compare their associations with hypertension relative to HOMA-IR, a well-established indicator of insulin resistance.

Analyses were performed with R (version 4.3.1) and Free Statistics software (version 2.1, Beijing, China^1^). *P* < 0.05 (two-sided) was defined as statistically significant.

## Results

### Study population characteristics

The current study included 4,302 postmenopausal women (mean age 64.1 ± 11.6 years), of whom 2,826 (65.7%) were diagnosed with hypertension. As delineated in Table 1, marked epidemiological and phenotypic divergences were identified through rigorous parametric comparisons between the hypertensive and non-hypertensive groups. Compared with non-hypertensive participants, those with hypertension were older and disproportionately identified as Non-Hispanic Black, less educated, lower family income, and more prone to residing alone. Those who developed hypertension had lower rates of current smoking and alcohol consumption, along with significantly less weekly physical activity; had higher HbA1c, uric acid levels, TyG index, and TyG-related indices; had a slightly later median age at menopause; and had no difference in hormone replacement therapy utilization.

**TABLE 1:** Baseline characteristics of the study participants.

Variables	Total (*n* = 4,302)	Non-hypertension (*n* = 1,476)	Hypertension (*n* = 2,826)	*P-value*
Age, mean ± SD, year	64.1 ± 11.6	59.5 ± 11.6	66.5 ± 10.9	< 0.001
Race, *n* (%)				< 0.001
Mexican American	2,242 (52.1)	854 (57.9)	1,388 (49.1)	–
Non-Hispanic Black	828 (19.2)	167 (11.3)	661 (23.4)	–
Non-Hispanic White	672 (15.6)	246 (16.7)	426 (15.1)	–
Other Hispanic	339 (7.9)	129 (8.7)	210 (7.4)	–
Other	221 (5.1)	80 (5.4)	141 (5.0)	–
Marital status, *n* (%)				< 0.001
Married/living with partner	2,184 (51.4)	843 (57.8)	1,341 (48.0)	–
Living alone	2,069 (48.6)	616 (42.2)	1,453 (52.0)	–
PIR, *n* (%)				< 0.001
≤ 1.3	1,116 (28.7)	320 (24.1)	796 (31.1)	–
1.3∼3.5	1590 (40.9)	510 (38.5)	1,080 (42.2)	–
> 3.5	1,178 (30.3)	496 (37.4)	682 (26.7)	–
Education level, *n* (%)				< 0.001
Less than high school	1,297 (30.2)	391 (26.5)	906 (32.1)	–
High school or equivalent	1,091 (25.4)	355 (24.1)	736 (26.1)	–
Above high school	1,906 (44.4)	729 (49.4)	1,177 (41.8)	–
Smoking status, *n* (%)				< 0.001
Never	2,496 (58.1)	813 (55.2)	1,683 (59.6)	–
Former	1,147 (26.7)	372 (25.2)	775 (27.5)	–
Current	653 (15.2)	289 (19.6)	364 (12.9)	–
Drinking status, *n* (%)				< 0.001
Never	977 (22.7)	278 (18.8)	699 (24.8)	–
Former	1,115 (26.0)	325 (22.0)	790 (28.0)	–
Current	2,204 (51.3)	872 (59.1)	1,332 (47.2)	–
Physical activity, median (IQR), minutes/week	60.0 (0.0, 283.5)	94.5 (0.0, 360.0)	31.5 (0.0, 240.0)	< 0.001
LDL-C, mean ± SD, mmol/L	3.2 ± 1.0	3.3 ± 0.9	3.1 ± 1.0	< 0.001
HDL-C, mean ± SD, mmol/L	1.5 ± 0.4	1.6 ± 0.4	1.5 ± 0.4	0.012
TC, mean ± SD, mmol/L	5.4 ± 1.1	5.5 ± 1.0	5.4 ± 1.1	< 0.001
HbA1c, mean ± SD, (%)	5.9 ± 1.1	5.7 ± 0.9	6.0 ± 1.2	< 0.001
Uric acid, mean ± SD, umol/L	314.1 ± 82.7	286.1 ± 66.8	328.8 ± 86.4	< 0.001
Menopausal age, median (IQR), year	46.0 (39.0, 51.0)	46.0 (39.0, 50.0)	46.0 (39.0, 52.0)	0.031
Hormone replacement therapy, *n* (%)				0.933
No	2,466 (57.3)	847 (57.4)	1,619 (57.3)	–
Yes	1,835 (42.7)	628 (42.6)	1,207 (42.7)	–
TyG index, mean ± SD	8.8 ± 0.6	8.7 ± 0.6	8.9 ± 0.6	< 0.001
TyG-BMI, mean ± SD	261.6 ± 66.1	244.7 ± 58.9	270.4 ± 67.9	< 0.001
TyG-WC, mean ± SD	873.8 ± 161.0	826.7 ± 153.0	898.4 ± 159.6	< 0.001
TyG-WHtR, mean ± SD	5.5 ± 1.0	5.2 ± 1.0	5.7 ± 1.0	< 0.001

Data are presented as medians (interquartile range), median (IQR), or *n* (%). PIR, Poverty Income Ratio; HDL-C, high-density lipoprotein cholesterol; LDL-C, low-density lipoprotein cholesterol; TC, total cholesterol; HbA1c, glycosylated hemoglobin; TyG, triglyceride glucose; BMI, body mass index; WC, waist circumference; WHtR, waist-to-height ratio.

### Association between TyG-associated indices and hypertension in postmenopausal women

**FIGURE 2:**
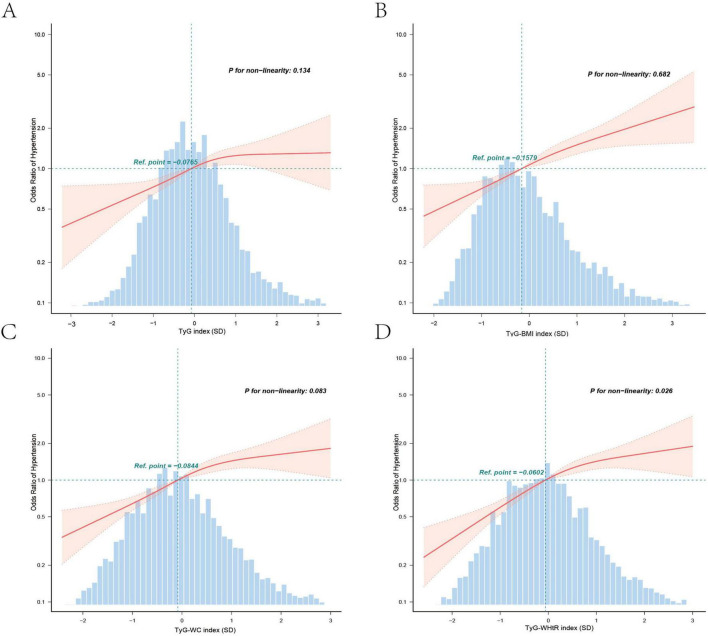
Association between TyG-associated indices and odds ratio for hypertension in postmenopausal women. Graphs (A–D) represent the TyG index, TyG-BMI index, TyG-WC index, and TyG-WHtR index, respectively. Solid and dashed lines represent the predicted value and 95% confidence intervals. They were adjusted for National Health and Nutrition Examination Survey (NHANES) cycles, age, race/ethnicity, marital status, education level, PIR, smoking status, drinking status, physical activity, HDL-C, LDL-C, glycosylated hemoglobin (HbA1c), uric acid, menopausal age, and hormone replacement therapy. Only 99.5% of the data is shown. TyG, triglyceride-glucose; BMI, body mass index; WC, waist circumference; WHtR, waist-to-height ratio; OR, odds ratio; 95% CI, 95% confidence interval; PIR, poverty income ratio; HDL-C, high-density lipoprotein cholesterol; LDL-C, low-density lipoprotein cholesterol.

Covariate-adjusted logistic regression results are presented in Tables 2, 3. After full adjustment (Model 4), each SD increase in the TyG index (OR = 1.33, 95% CI: 1.18–1.49), TyG-BMI (OR = 1.42, 95% CI: 1.28–1.57), TyG-WC (OR = 1.42, 95% CI: 1.28–1.58), and TyG-WHtR (OR = 1.49, 95% CI: 1.34–1.66) remained significantly associated with elevated hypertension risk (all *P* < 0.001). Quartile analyses revealed graded relationships: compared to T1, T3 of TyG-WHtR exhibited the highest risk (OR = 2.13, 95% CI: 1.69–2.68), followed by TyG-WC (OR = 2.11, 95% CI: 1.68–2.65), TyG-BMI (OR = 1.95, 95% CI: 1.56–2.44), and TyG index (OR = 1.52, 95% CI: 1.20–1.92). All trend *P*-values were < 0.001, indicating robust linear associations. The RCS curve delineated a J-shaped curve association between TyG-WHtR (SD) and hypertension risk (P for non-linearity = 0.026) (Figure 2D), whereas TyG index (SD), TyG-BMI (SD), and TyG-WC (SD) displayed near-linear trends (P for non-linearity > 0.05) (Figures 2A–C).

**TABLE 2:** Association between TyG-associated indices and hypertension in postmenopausal women.

Variables	Model 1	*P*	Model 2	*P*	Model 3	*P*	Model 4	*P*
TyG index (per SD)	1.37 (1.28∼1.47)	< 0.001	1.44 (1.33∼1.56)	< 0.001	1.44 (1.33∼1.56)	< 0.001	1.33 (1.18∼1.49)	< 0.001
TyG-BMI (per SD)	1.54 (1.44∼1.65)	< 0.001	1.66 (1.53∼1.81)	< 0.001	1.66 (1.53∼1.81)	< 0.001	1.42 (1.28∼1.57)	< 0.001
TyG-WC (per SD)	1.63 (1.52∼1.74)	< 0.001	1.64 (1.51∼1.77)	< 0.001	1.63 (1.50∼1.77)	< 0.001	1.42 (1.28∼1.58)	< 0.001
TyG-WHtR (per SD)	1.71 (1.59∼1.83)	< 0.001	1.69 (1.56∼1.84)	< 0.001	1.69 (1.55∼1.83)	< 0.001	1.49 (1.34∼1.66)	< 0.001

Model 1 was adjusted for none; Model 2 was adjusted for NHANES cycles, age, race/ethnicity, marital status, education level, and PIR; Model 3 was adjusted for Model 2 plus smoking status, drinking status, and physical activity; Model 4 was adjusted for Model 3 plus HDL-C, LDL-C, HbA1c, uric acid, menopausal age, and hormone replacement therapy. TyG, triglyceride-glucose; BMI, body mass index; WC, waist circumference; WHtR, waist-to-height ratio; SD, standard deviation; OR, odds ratio; 95% CI, 95% confidence interval; PIR, poverty income ratio; HDL-C, high-density lipoprotein cholesterol; LDL-C, low-density lipoprotein cholesterol.

**TABLE 3:** Association between tertiles of TyG-associated indices and hypertension in postmenopausal women.

Variables	Model 1	*P*	Model 2	*P*	Model 3	*P*	Model 4	*P*
**TyG index**								
T 1	1 (ref)	–	1 (ref)	–	1 (ref)	–	1 (ref)	–
T 2	1.51 (1.29∼1.75)	< 0.001	1.45 (1.21∼1.73)	< 0.001	1.46 (1.23∼1.75)	< 0.001	1.30 (1.07∼1.57)	< 0.001
T 3	1.90 (1.63∼2.23)	< 0.001	2.06 (1.71∼2.47)	< 0.001	2.06 (1.71∼2.48)	< 0.001	1.52 (1.20∼1.92)	< 0.001
P for trend	–	< 0.001	–	< 0.001	–	< 0.001	–	< 0.001
**TyG-BMI**								
T 1	1 (ref)	–	1 (ref)	–	1 (ref)	–	1 (ref)	–
T 2	1.69 (1.45∼1.96)	< 0.001	1.65 (1.39∼1.97)	< 0.001	1.65 (1.38∼1.97)	< 0.001	1.40 (1.16∼1.70)	< 0.001
T 3	2.51 (2.14∼2.95)	< 0.001	2.93 (2.43∼3.52)	< 0.001	2.88 (2.39∼3.48)	< 0.001	1.95 (1.56∼2.44)	< 0.001
P for trend	–	< 0.001	–	< 0.001	–	< 0.001	–	< 0.001
**TyG-WC**								
T 1	1 (ref)	–	1(Ref)	–	1(Ref)	–	1(Ref)	–
T 2	1.99 (1.71∼2.32)	< 0.001	1.89 (1.59∼2.26)	< 0.001	1.89 (1.58∼2.25)	< 0.001	1.73 (1.43∼2.09)	< 0.001
T 3	2.94 (2.51∼3.45)	< 0.001	3.08 (2.56∼3.71)	< 0.001	3.04 (2.52∼3.66)	< 0.001	2.11 (1.68∼2.65)	< 0.001
P for trend	–	< 0.001	–	< 0.001	–	< 0.001	–	< 0.001
**TyG-WHtR**								
T 1	1 (ref)	–	1 (ref)	–	1 (ref)	–	1 (ref)	–
T 2	2.00 (1.72∼2.33)	< 0.001	1.84 (1.54∼2.19)	< 0.001	1.83 (1.53∼2.18)	< 0.001	1.65 (1.36∼1.99)	< 0.001
T 3	3.08 (2.62∼3.61)	< 0.001	3.06 (2.54∼3.69)	< 0.001	3.03 (2.51∼3.66)	< 0.001	2.13 (1.69∼2.68)	< 0.001
P for trend	–	< 0.001	–	< 0.001	–	< 0.001	–	< 0.001

Model 1 was adjusted for none; Model 2 was adjusted for NHANES cycles, age, race/ethnicity, marital status, education level, and PIR; Model 3 was adjusted for Model 2 plus smoking status, drinking status, and physical activity; Model 4 was adjusted for Model 3 plus HDL-C, LDL-C, HbA1c, uric acid, menopausal age, and hormone replacement therapy. TyG, triglyceride-glucose; BMI, body mass index; WC, waist circumference; WHtR, waist-to-height ratio; SD, standard deviation; OR, odds ratio; 95% CI, 95% confidence interval; T, tertile; PIR, poverty income ratio; HDL-C, high-density lipoprotein cholesterol; LDL-C, low-density lipoprotein cholesterol.

### ROC curve between TyG index and its combined obesity index and the prevalence of hypertension

**FIGURE 3:**
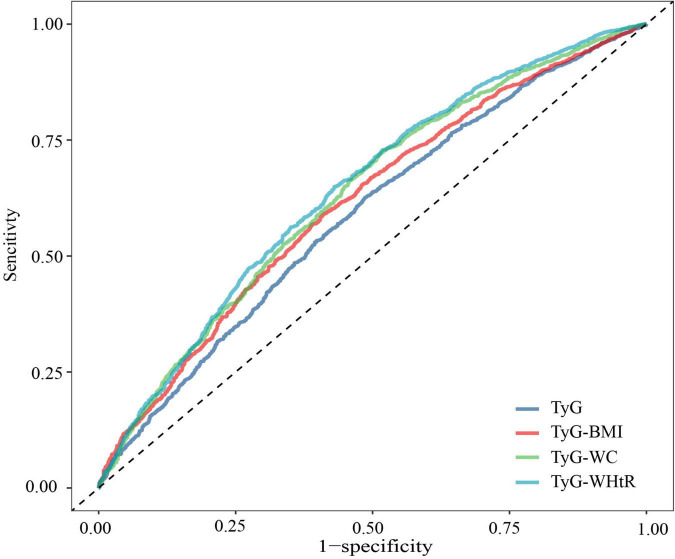
Receiver operating characteristic (ROC) curve between triglyceride-glucose (TyG) index and its combined obesity index and the risk of hypertension.

Receiver operating characteristic analysis evaluating the TyG index and its obesity-integrated derivatives against hypertension prevalence revealed distinct discriminatory abilities. TyG-WHtR showed superior predictive validity (AUC = 0.643, 95% CI 0.625–0.660), outperforming TyG-WC (AUC = 0.632, 0.614–0.650) and TyG-BMI (AUC = 0.615, 0.597–0.632). The stand-alone TyG index showed a comparatively lower diagnostic precision (AUC = 0.588, 0.570–0.606) (Figure 3).

### Sensitivity analysis

In order to assess the robustness of the findings, sensitivity analyses were performed using the complete-case dataset (pre-imputation). The associations between TyG-associated indices (TyG index, TyG-BMI, TyG-WC, and TyG-WHtR) and hypertension remained statistically significant across all multivariable-adjusted models, both for continuous and categorical (tertile-based) analyses. These results closely aligned with the primary analyses derived from multiply imputed data (Supplementary Tables 1, 2).

Furthermore, sensitivity analyses using multivariate regression and ROC curves demonstrated that the TyG index, its obesity indicators (TyG-BMI, TyG-WC, and TyG-WHtR), and HOMA-IR were significantly associated with hypertension risk. Notably, the TyG-WHtR index exhibited superior discriminative capacity for hypertension, with an AUC of 0.643 (95% CI: 0.626–0.661), compared to HOMA-IR (AUC = 0.630, 95% CI: 0.612–0.647). Detailed results of these analyses are provided in Supplementary Tables 3, 4, while the ROC analysis findings are summarized in Supplementary Figure 1 and Supplementary Table 5.

## Discussion

This population-based study focused on postmenopausal women to delineate the relationships between the TyG index, integrated adiposity parameters, and hypertension prevalence. Quantitative analyses revealed that elevated TyG levels in combination with obesity parameters, especially TyG-WHtR, exhibited a dose-dependent association with hypertension prevalence. Of particular note was the non-linear relationship observed between TyG-WHtR and hypertension risk, in contrast to the linear patterns shown by other composite markers. The ROC analysis revealed that TyG-WHtR was the most robust predictor, outperforming conventional obesity indicators.

The hallmark of IR is the attenuation of insulin signaling efficacy across target organs, which culminates in systemic impairments in glucose absorption and cellular energy conversion pathways (22). A substantial body of research has established a strong correlation between IR and the development of hypertension (23–25). Postmenopausal women have been shown to have a significantly higher incidence of IR along with decreased insulin sensitivity (26). This phenomenon is widely believed to be related to the decline in estrogen levels that accompanies the menopausal transition (27).

Conventional insulin resistance assessment methods present inherent limitations, ranging from technical complexity to biomarker dependency. Recent studies have indicated that the TyG index, when combined with anthropometric parameters, may offer a pragmatic solution, providing diagnostic accuracy comparable to conventional measures while reducing operational costs. This renders it a promising instrument for the evaluation of IR (28). Despite the link between these metrics and hypertension have been widely explored (10, 29), research focusing on postmenopausal women remains limited. Ben Ali S et al. (4) demonstrated that WC, apoB, and HOMA-IR were the strongest risk factors for predicting postmenopausal hypertension in women aged 35–70 years. Ding et al. (30) reported that elevated TyG levels serve as an independent predictor of H-type hypertension development among women undergoing menopausal transition. Notably, RCS models confirmed a positive linear correlation (P for non-linearity = 0.866). Additionally, in a cross-sectional study conducted by Choi et al. (31), post-menopausal women in South Korea with a higher HOMA-IR were more likely to be in the hypertensive group (RRR = 4.37, *P* < 0.001). Similarly, the present analyses validated TyG as an independent risk factor for hypertension in postmenopausal women. Our results also showed that TyG-BMI, TyG-WC, and TyG-WHtR were positively associated with the risk of hypertension in postmenopausal women; the AUC [0.643 (95% CI: 0.625–0.660)] of TyG-WHtR was higher than other obesity composites in predicting hypertension. Which was consistent with the results of a cross-sectional study from Chinese population-level investigations, where TyG-WHtR demonstrated superior discriminative ability for hypertension in all individuals (32). Complementary evidence from a study of the China Health and Nutrition Survey confirmed this finding, with heightened efficacy observed in female subgroups (11).

Numerous studies have indicated that measures of abdominal obesity have superior predictive validity for obesity-related cardiometabolic risk associated with obesity in comparison to BMI. This is due to the fact that the visceral fat layer located in the abdominal region exhibits elevated metabolic and inflammatory activity in contrast to the subcutaneous fat layer found in other anatomical areas (33, 34). Ashwell et al. (35) demonstrated that WHtR was considered a more effective measure for identifying abdominal obesity compared to BMI and WC. In addition, WHtR was found to be a better predictor of the risk of diabetes, dyslipidaemia, hypertension, and cardiovascular disease than WC in populations of different nationalities and ethnicities. Huang et al. (36) demonstrated TyG-WHtR was associated with the risk of hypertension (OR = 1.12, 95% CI: 1.11–1.14, *P* < 0.001), and TyG-WHtR had the best predictive performance for hypertension in United States adults aged 18–60. It has been demonstrated that postmenopausal women are susceptible to abdominal obesity and IR due to the hormonal changes in the body (4). The results of this study further support that TyG-WHtR is a better predictor of hypertension than TyG-WC and TyG-BMI in this high-risk population. Furthermore, the result of the ROC model highlights the key role of TyG-WHtR in the progression of hypertension in postmenopausal women.

From a mechanistic perspective, as a core pathophysiological driver of metabolic syndrome, IR establishes intricate mechanistic links with multisystem dysregulation. These include endothelial dysfunction, dyslipidemia, sympathetic overactivity, and chronic low-grade inflammation, which collectively accelerate the pathogenesis of hypertension. In the menopausal transition, due to the estrogen depletion, this phenomenon is also associated with postmenopausal weight changes, accumulation of abdominal fat, increased plasma endothelin levels, overproduction of reactive oxygen species, and increased sympathetic activity, which may together heighten the risk of hypertension in this population (37).

Although the current large cross-sectional study offers compelling evidence for the relationship between the TyG index and its adiposity composites (especially TyG-WHtR) and risk stratification for hypertension in postmenopausal women, some limitations should be acknowledged. First, the observational nature of this cross-sectional study limits conclusions about causality. Second, despite extensive covariate adjustment in the multivariate analyses, unmeasured or unknown residual confounders (e.g., dietary habits, antihypertensive medication) may introduce residual bias. Third, although the study used nationally representative data, the limitation to United States residents requires caution when extrapolating to other populations. Therefore, caution should be exercised in generalizing these findings to other populations, especially for races underrepresented in the sampling frame.

## Conclusion

The TyG index and its combined obesity indicators, especially TyG-WHtR, are strongly associated with the prevalence of hypertension in postmenopausal women. TyG-WHtR may be a valuable biomarker for targeted screening in this population.

## Data Availability

Publicly available datasets were analyzed in this study. This data can be found here: https://www.cdc.gov/nchs/nhanes/.

## Footnotes

1
http://www.clinicalscientists.cn/freestatistics
