# Supplementary material for: Association between triglyceride-glucose index and its obesity indicators with hypertension in postmenopausal women: a cross-sectional study
Source: Front Nutr. 2025 Jul 17;12:1623697. doi: 10.3389/fnut.2025.1623697 (PMC12310447; doi:10.3389/fnut.2025.1623697)
Supplement: Supplementary file 1 [file Data_Sheet_1.docx]

**Supplementary Materials Files**

To: **Association between triglyceride-glucose index and its obesity indicators with hypertension in postmenopausal women: A cross-sectional study**

Bo Zhang^1†^ Daoli Jiang^1†^ He Ma^2 ǂ^ Huanxian Liu^3 *^

**Supplementary Method**

***Multiple imputation***

Multiple imputation is a robust and widely used statistical technique for handling missing data across various fields, including epidemiology, social sciences, and clinical research. The method involves generating several plausible datasets through imputation, conducting separate analyses on each dataset, and pooling the results to produce valid estimates and measures of uncertainty [1, 2]. This technique helps mitigate potential biases and ensures the representativeness of the findings.

The multiple imputation by chained equations (MICE) method, employed in this study, is a flexible approach that sequentially imputes missing values for each variable by developing regression models conditioned on the observed values of other variables [1, 2]. We created three imputed datasets and combined the results according to Rubin’s Rules, ensuring the robustness and reliability of our conclusions.

**References:**

1. White IR, Royston P, Wood AM (2011) Multiple imputation using chained equations: Issues and guidance for practice. Stat Med 30:377–399. https://doi.org/10.1002/sim.4067

2 Beesley LJ, Bondarenko I, Elliot MR, et al (2021) Multiple imputation with missing data indicators. Stat Methods Med Res 30:2685–2700. <https://doi.org/10.1177/09622802211047346>

**Supplementary Table**

Table S1: Association between TyG-associated indices and hypertension in postmenopausal women (pre-imputation).

Table S2: Association between tertiles of TyG-associated indices and hypertension in postmenopausal women (pre-imputation).

Table S3. Association of TyG-related indices and HOMA-IR with hypertension in postmenopausal women (after-imputation).

Table S4. Association of TyG-related indices and HOMA-IR with hypertension in postmenopausal women (after-imputation).

Table S5. Discriminative Performance of TyG-Related Indices and HOMA-IR for Hypertension in Postmenopausal Women.

Figure S1. ROC Curve: Comparative Analysis of TyG-Related Indices and HOMA-IR in Predicting Hypertension Risk.

**Table S1.** Association between TyG-associated indices and hypertension in postmenopausal women : Complete-case analysis (pre-imputation).

| **Variables** | **Model 1** | ***P*** | **Model 2** | ***P*** | **Model 3** | ***P*** | **Model 4** | ***P*** |
| --- | --- | --- | --- | --- | --- | --- | --- | --- |
| **TyG index**  **(Per SD)** | 1.37 (1.28~1.47) | < 0.001 | 1.43 (1.32~1.54) | < 0.001 | 1.43 (1.33~1.55) | < 0.001 | 1.29 (1.16~1.42) | < 0.001 |
| **TyG-BMI**  **(Per SD)** | 1.54 (1.44~1.65) | < 0.001 | 1.67 (1.55~1.81) | < 0.001 | 1.67 (1.54~1.81) | < 0.001 | 1.44 (1.31~1.58) | < 0.001 |
| **TyG-WC**  **(Per SD)** | 1.63 (1.52~1.74) | < 0.001 | 1.65 (1.53~1.78) | < 0.001 | 1.65 (1.53~1.78) | < 0.001 | 1.45 (1.32~1.60) | < 0.001 |
| **TyG-WHtR**  **(Per SD)** | 1.71 (1.59~1.83) | < 0.001 | 1.70 (1.57~1.84) | < 0.001 | 1.70 (1.57~1.84) | < 0.001 | 1.51 (1.37~1.67) | < 0.001 |

**Notes:** Model 1 was adjusted for none; Model 2 was adjusted for NHANES cycles, age, race/ethnicity, marital status, education level, and PIR; Model 3 was adjusted for Model 2 plus smoking status, drinking status, and physical activity; Model 4 was adjusted for Model 3 plus HDL-C, LDL-C, HbA1c, uric acid, menopausal age, and hormone replacement therapy.

**Abbreviations:** TyG, triglyceride-glucose; BMI, body mass index; WC, waist circumference; WHtR, waist-to-height ratio; SD, standard deviation; OR, odds ratio; 95% CI, 95% confidence interval; PIR, poverty income ratio; HDL-C, high-density lipoprotein cholesterol; LDL-C, low-density lipoprotein cholesterol.

**Table S2.** Association between tertiles of TyG-associated indices and hypertension in postmenopausal women : Complete-case analysis (pre-imputation).

| **Variables** | **Model 1** | ***P*** | **Model 2** | ***P*** | **Model 3** | ***P*** | **Model 4** | ***P*** |
| --- | --- | --- | --- | --- | --- | --- | --- | --- |
| **TyG index** |  |  |  |  |  |  |  |  |
| **T 1** | 1(Ref) |  | 1(Ref) |  | 1(Ref) |  | 1(Ref) |  |
| **T 2** | 1.51 (1.29~1.75) | < 0.001 | 1.47 (1.25~1.74) | < 0.001 | 1.48 (1.25~1.75) | < 0.001 | 1.31 (1.10~1.57) | < 0.001 |
| **T3** | 1.90 (1.63~2.23) | < 0.001 | 2.06 (1.73~2.44) | < 0.001 | 2.06 (1.73~2.45) | < 0.001 | 1.52 (1.23~1.88) | < 0.001 |
| **P for trend** |  | < 0.001 |  | < 0.001 |  | < 0.001 |  | < 0.001 |
| **TyG-BMI** |  |  |  |  |  |  |  |  |
| **T 1** | 1(Ref) |  | 1(Ref) |  | 1(Ref) |  | 1(Ref) |  |
| **T 2** | 1.69 (1.45~1.96) | < 0.001 | 1.68 (1.42~1.98) | < 0.001 | 1.68 (1.42~1.99) | < 0.001 | 1.44 (1.21~1.72) | < 0.001 |
| **T3** | 2.51 (2.14~2.95) | < 0.001 | 2.98 (2.50~3.56) | < 0.001 | 2.95 (2.47~3.53) | < 0.001 | 2.05 (1.66~2.52) | < 0.001 |
| **P for trend** |  | < 0.001 |  | < 0.001 |  | < 0.001 |  | < 0.001 |
| **TyG-WC** |  |  |  |  |  |  |  |  |
| **T 1** | 1(Ref) |  | 1(Ref) |  | 1(Ref) |  | 1(Ref) |  |
| **T 2** | 1.99 (1.71~2.32) | < 0.001 | 1.89 (1.60~2.23) | < 0.001 | 1.88 (1.59~2.22) | < 0.001 | 1.69 (1.42~2.02) | < 0.001 |
| **T3** | 2.94 (2.51~3.45) | < 0.001 | 3.16 (2.66~3.77) | < 0.001 | 3.14 (2.63~3.74) | < 0.001 | 2.27 (1.83~2.80) | < 0.001 |
| **P for trend** |  | < 0.001 |  | < 0.001 |  | < 0.001 |  | < 0.001 |
| **TyG-WHtR** |  |  |  |  |  |  |  |  |
| **T 1** | 1(Ref) |  | 1(Ref) |  | 1(Ref) |  | 1(Ref) |  |
| **T 2** | 2.00 (1.72~2.33) | < 0.001 | 1.86 (1.57~2.19) | < 0.001 | 1.85 (1.57~2.19) | < 0.001 | 1.64 (1.37~1.96) | < 0.001 |
| **T3** | 3.08 (2.62~3.61) | < 0.001 | 3.15 (2.63~3.75) | < 0.001 | 3.12 (2.61~3.72) | < 0.001 | 2.26 (1.82~2.79) | < 0.001 |
| **P for trend** |  | < 0.001 |  | < 0.001 |  | < 0.001 |  | < 0.001 |

**Notes:** Model 1 was adjusted for none; Model 2 was adjusted for NHANES cycles, age, race/ethnicity, marital status, education level, and PIR; Model 3 was adjusted for Model 2 plus smoking status, drinking status, and physical activity; Model 4 was adjusted for Model 3 plus HDL-C, LDL-C, HbA1c, uric acid, menopausal age, and hormone replacement therapy.

**Abbreviations:** TyG, triglyceride-glucose; BMI, body mass index; WC, waist circumference; WHtR, waist-to-height ratio; SD, standard deviation; OR, odds ratio; 95% CI, 95% confidence interval; T, tertiles; PIR, poverty income ratio; HDL-C, high-density lipoprotein cholesterol; LDL-C, low-density lipoprotein cholesterol.

**Table S3.** Association of TyG-related indices and HOMA-IR with hypertension in postmenopausal women (after-imputation).

| **Variables** | **Model 1** | ***P*** | **Model 2** | ***P*** | **Model 3** | ***P*** | **Model 4** | ***P*** |
| --- | --- | --- | --- | --- | --- | --- | --- | --- |
| **TyG index**  **(Per SD)** | 1.38 (1.29~1.48) | < 0.001 | 1.47 (1.37~1.59) | < 0.001 | 1.48 (1.37~1.59) | < 0.001 | 1.38 (1.25~1.52) | < 0.001 |
| **TyG-BMI**  **(Per SD)** | 1.54 (1.43~1.65) | < 0.001 | 1.48 (1.37~1.59) | < 0.001 | 1.44 (1.34~1.55) | < 0.001 | 1.23 (1.12~1.34) | < 0.001 |
| **TyG-WC**  **(Per SD)** | 1.63 (1.52~1.75) | < 0.001 | 1.57 (1.46~1.69) | < 0.001 | 1.55 (1.44~1.66) | < 0.001 | 1.35 (1.23~1.48) | < 0.001 |
| **TyG-WHtR**  **(Per SD)** | 1.71 (1.59~1.84) | < 0.001 | 1.69 (1.56~1.82) | < 0.001 | 1.65 (1.53~1.78) | < 0.001 | 1.47 (1.34~1.62) | < 0.001 |
| **HOMA-IR**  **(Per SD)** | 1.90 (1.63~2.21) | < 0.001 | 1.74 (1.50~2.02) | < 0.001 | 1.66 (1.43~1.92) | < 0.001 | 1.20 (1.04~1.38) | 0.012 |

**Notes:** Model 1 was adjusted for none; Model 2 was adjusted for NHANES cycles, age, race/ethnicity, marital status, education level, and PIR; Model 3 was adjusted for Model 2 plus smoking status, drinking status, and physical activity; Model 4 was adjusted for Model 3 plus HDL-C, LDL-C, HbA1c, uric acid, menopausal age, and hormone replacement therapy.

**Abbreviations:** TyG, triglyceride-glucose; BMI, body mass index; WC, waist circumference; WHtR, waist-to-height ratio; HOMA-IR, homeostasis model assessment of insulin resistance; SD, standard deviation; OR, odds ratio; 95% CI, 95% confidence interval; PIR, poverty income ratio; HDL-C, high-density lipoprotein cholesterol; LDL-C, low-density lipoprotein cholesterol.

**Table S4.** Association of TyG-related indices and HOMA-IR with hypertension in postmenopausal women (after-imputation).

| **Variables** | **Model 1** | ***P*** | **Model 2** | ***P*** | **Model 3** | ***P*** | **Model 4** | ***P*** |
| --- | --- | --- | --- | --- | --- | --- | --- | --- |
| **TyG index** |  |  |  |  |  |  |  |  |
| **T 1** | 1(Ref) |  | 1(Ref) |  | 1(Ref) |  | 1(Ref) |  |
| **T 2** | 1.51 (1.30~1.76) | < 0.001 | 1.66 (1.41~1.95) | < 0.001 | 1.67 (1.42~1.96) | < 0.001 | 1.52 (1.27~1.81) | < 0.001 |
| **T3** | 1.91 (1.63~2.23) | < 0.001 | 2.15 (1.82~2.54) | < 0.001 | 2.17 (1.83~2.57) | < 0.001 | 1.73 (1.40~2.13) | < 0.001 |
| **P for trend** |  | < 0.001 |  | < 0.001 |  | < 0.001 |  | < 0.001 |
| **TyG-BMI** |  |  |  |  |  |  |  |  |
| **T 1** | 1(Ref) |  | 1(Ref) |  | 1(Ref) |  | 1(Ref) |  |
| **T 2** | 1.69 (1.45~1.96) | < 0.001 | 1.58 (1.35~1.85) | < 0.001 | 1.55 (1.32~1.81) | < 0.001 | 1.32 (1.11~1.56) | 0.002 |
| **T3** | 2.49 (2.12~2.92) | < 0.001 | 2.26 (1.92~2.67) | < 0.001 | 2.15 (1.82~2.55) | < 0.001 | 1.49 (1.23~1.82) | <0.001 |
| **P for trend** |  | < 0.001 |  | < 0.001 |  | < 0.001 |  | < 0.001 |
| **TyG-WC** |  |  |  |  |  |  |  |  |
| **T 1** | 1(Ref) |  | 1(Ref) |  | 1(Ref) |  | 1(Ref) |  |
| **T 2** | 2.01 (1.72~2.34) | < 0.001 | 1.91 (1.63~2.24) | < 0.001 | 1.86 (1.59~2.19) | < 0.001 | 1.68 (1.42~2.00) | < 0.001 |
| **T3** | 2.97 (2.53~3.48) | < 0.001 | 2.78 (2.35~3.28) | < 0.001 | 2.68 (2.27~3.17) | < 0.001 | 1.95 (1.59~2.39) | < 0.001 |
| **P for trend** |  | < 0.001 |  | < 0.001 |  | < 0.001 |  | < 0.001 |
| **TyG-WHtR** |  |  |  |  |  |  |  |  |
| **T 1** | 1(Ref) |  | 1(Ref) |  | 1(Ref) |  | 1(Ref) |  |
| **T 2** | 2.01 (1.72~2.34) | < 0.001 | 1.95 (1.67~2.29) | < 0.001 | 1.89 (1.61~2.21) | < 0.001 | 1.67 (1.41~1.99) | < 0.001 |
| **T3** | 3.08 (2.62~3.62) | < 0.001 | 2.97 (2.51~3.52) | < 0.001 | 2.82 (2.38~3.35) | < 0.001 | 2.08 (1.69~2.55) | < 0.001 |
| **P for trend** |  | < 0.001 |  | < 0.001 |  | < 0.001 |  | < 0.001 |
| **HOMA-IR** |  |  |  |  |  |  |  |  |
| **T 1** | 1(Ref) |  | 1(Ref) |  | 1(Ref) |  | 1(Ref) |  |
| **T 2** | 1.74 (1.49~2.02) | < 0.001 | 1.65 (1.41~1.93) | < 0.001 | 1.61 (1.38~1.89) | < 0.001 | 1.45 (1.22~1.71) | < 0.001 |
| **T3** | 2.73 (2.32~3.20) | < 0.001 | 2.50 (2.11~2.95) | < 0.001 | 2.37 (2.00~2.81) | < 0.001 | 1.62 (1.33~1.98) | < 0.001 |
| **P for trend** |  | < 0.001 |  | < 0.001 |  | < 0.001 |  | < 0.001 |

**Notes:** Model 1 was adjusted for none; Model 2 was adjusted for NHANES cycles, age, race/ethnicity, marital status, education level, and PIR; Model 3 was adjusted for Model 2 plus smoking status, drinking status, and physical activity; Model 4 was adjusted for Model 3 plus HDL-C, LDL-C, HbA1c, uric acid, menopausal age, and hormone replacement therapy.

**Abbreviations:** TyG, triglyceride-glucose; BMI, body mass index; WC, waist circumference; WHtR, waist-to-height ratio; HOMA-IR, homeostasis model assessment of insulin resistance; SD, standard deviation; OR, odds ratio; 95% CI, 95% confidence interval; PIR, poverty income ratio; HDL-C, high-density lipoprotein cholesterol; LDL-C, low-density lipoprotein cholesterol.

**Table S5.** Discriminative Performance of TyG-Related Indices and HOMA-IR for Hypertension in Postmenopausal Women

| **Variable** | **AUC** | **95% CI** |
| --- | --- | --- |
| TyG index | 0.589 | 0.571-0.607 |
| TYG-BMI | 0.615 | 0.597-0.663 |
| TYG-WC | 0.632 | 0.615-0.650 |
| TyG-WHtR | **0.643** | 0.626-0.661 |
| HOMA-IR | **0.630** | 0.612-0.647 |

**Abbreviations:** TyG, triglyceride-glucose; BMI, body mass index; WC, waist circumference; WHtR, waist-to-height ratio; HOMA-IR, homeostasis model assessment of insulin resistance; AUC, area under the curve; 95% CI, 95% confidence interval.


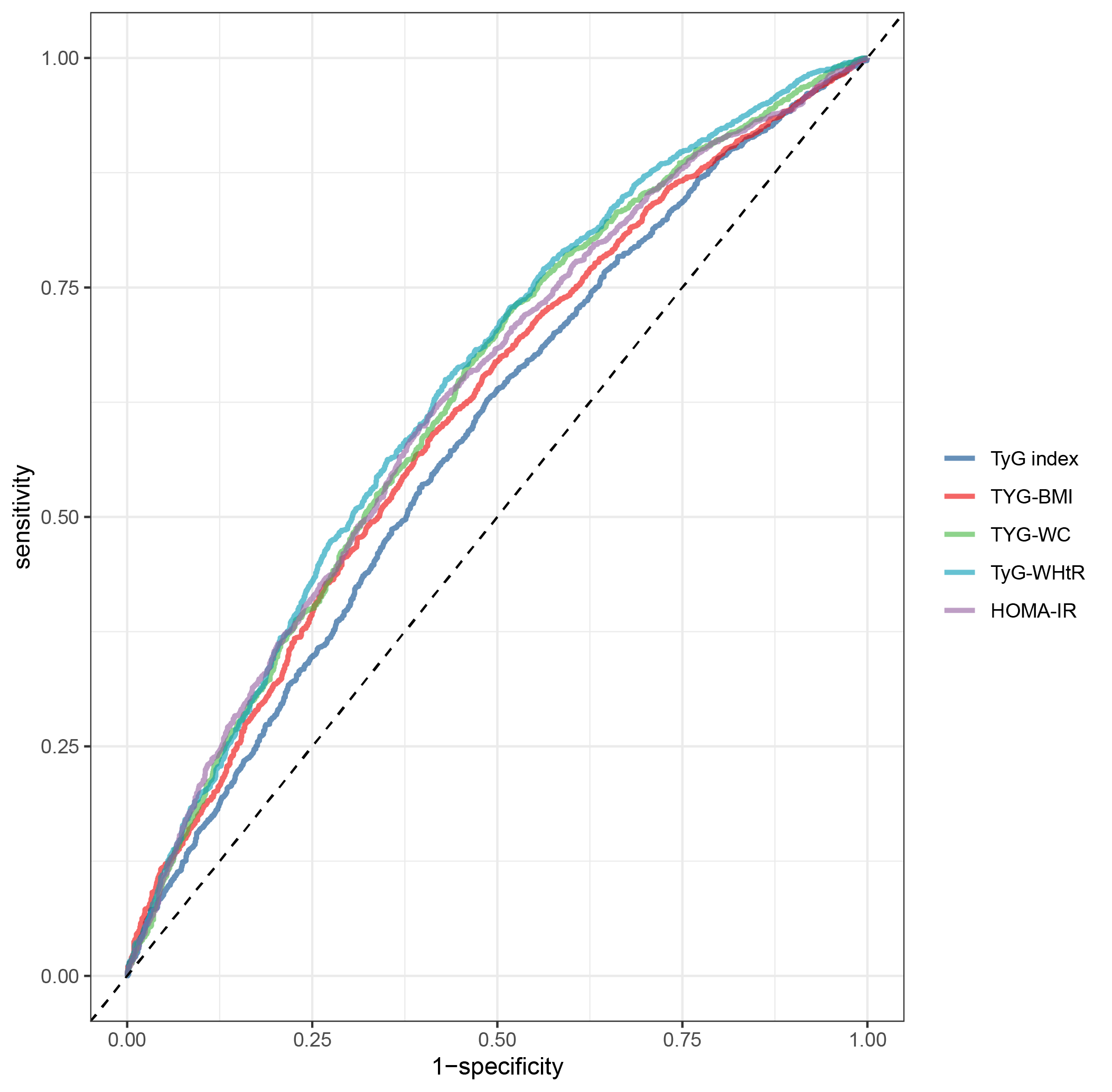


**Figure S1.** ROC Curve: Comparative Analysis of TyG-Related Indices and HOMA-IR in Predicting Hypertension Risk

**Abbreviations:** TyG, triglyceride-glucose; BMI, body mass index; WC, waist circumference; WHtR, waist-to-height ratio; HOMA-IR, homeostasis model assessment of insulin resistance.
